# *Ganab* Haploinsufficiency Does Not Cause Polycystic Kidney Disease or Polycystic Liver Disease in Mice

**DOI:** 10.1155/2020/7469428

**Published:** 2020-05-19

**Authors:** Guangrui Geng, Yunming Xiao, Yingjie Zhang, Wanjun Shen, Jiaona Liu, Fei Zhu, Xu Wang, Jie Wu, Ran Liu, Guangyan Cai, Xueyuan Bai, Qinggang Li, Xiangmei Chen

**Affiliations:** Medical School of Chinese PLA, Department of Nephrology, Chinese PLA General Hospital, Chinese PLA Institute of Nephrology, State Key Laboratory of Kidney Diseases, National Clinical Research Center for Kidney Diseases, Beijing, China

## Abstract

**Background:**

Heterozygous *GANAB* mutations that can cause autosomal dominant polycystic kidney disease (ADPKD) and polycystic liver disease (PLD) have been described previously, but their roles in ADPKD and PLD are largely unknown. With the increase in polycystic kidney disease caused by *GANAB* gene mutations in recent years, a suitable animal model is still needed to further explore the pathogenic role of this gene.

**Methods:**

To construct a mouse model of *Ganab* gene deletion, we analyzed *the Ganab* gene structure and designed two CRISPR-/Cas9-based targeting strategies. The Cas9/sgRNA we constructed was microinjected into fertilized mouse eggs to obtain chimeric F0 mice. Mice with stable genotypes were selected from offspring born after mating F0 mice with wild-type mice.

**Results:**

We found that homozygous mutation of the *Ganab* gene in C57BL/6 mice resulted in early embryonic lethality, and there were no cysts in the kidneys or livers of *Ganab*^+/-^ mice. Additionally, *Ganab* protein expression was reduced by at least 50%, while the expression of ADPKD proteins (PC1 and PC2) and acetylated tubulin was not affected in the *Ganab*^+/-^ kidney. However, the *Ganab*^+/-^ mice did not show any abnormal clinical phenotypes after birth and failed to reveal renal tubule dilatation or any abnormalities of the glomeruli in the *Ganab*^+/-^ kidney.

**Conclusions:**

Homozygous *Ganab* mutations are lethal in the fetal stage, and *Ganab* haploinsufficiency does not cause kidney or liver cysts in mice, suggesting that it may not be the causative gene in polycystic kidney disease.

## 1. Introduction

Several recent studies reported that autosomal dominant polycystic kidney disease (ADPKD) is mainly caused by mutations in the *PKD1* and *PKD2* genes and rarely by mutations in the *GANAB* gene [1]. *GANAB*, also known as *PKD3*, encodes the alpha subunit of the glucosidase II protein. Mutations in this gene can cause defects in protein maturation and cell surface localization of polycystin-1 and polycystin-2, resulting in polycystic kidney disease or polycystic liver disease [2]. Currently, all of the clinically identified human ADPKD patients with *GANAB* mutations are heterozygous mutants [3]. In general, leaky mutations can cause human diseases. Currently, there are more than 660 genes, including the *GANAB, PKD1*, and *PKD2* genes that cause diseases due to haploinsufficiency [4, 5].

To study the role of *Ganab* during cyst development in mice, we constructed two C57BL/6 mouse models with knockouts of different exonic regions of the *Ganab* gene via the clustered regularly interspaced short palindromic repeats (CRISPR)/Cas9 technique. However, we were surprised to find that there were no cysts in the kidneys or livers of *Ganab*^+/-^ mice constructed via two strategies. Furthermore, the *Ganab*^+/-^ mice did not show abnormal clinical phenotypes after birth, and renal ultrasound and renal angiography did not reveal obvious abnormalities in blood flow or blood vessels. We performed renal pathology examinations of all of the *Ganab*^+/-^ mice and failed to detect renal tubule dilatation or any abnormalities of the glomeruli. We also found that homozygous mutation of the *Ganab* gene (*Ganab*^−/−^) in C57BL/6 mice resulted in early embryonic lethality and the inability to survive past 3.5 days, while survival was not affected in *Ganab*^+/-^ mice. Our results suggest that heterozygous mutation or haploinsufficiency of *Ganab* does not result in polycystic kidney or polycystic liver disease in mice.

## 2. Materials and Methods

### 2.1. Subjects

C57BL/6 mice were purchased from Cyagen Biosciences Inc. and Biocytogen Biosciences Inc. All studies were reviewed by the Medical and Ethical Committee of the PLA General Hospital.

### 2.2. sgRNA Target Sequence

**Figure 1:**
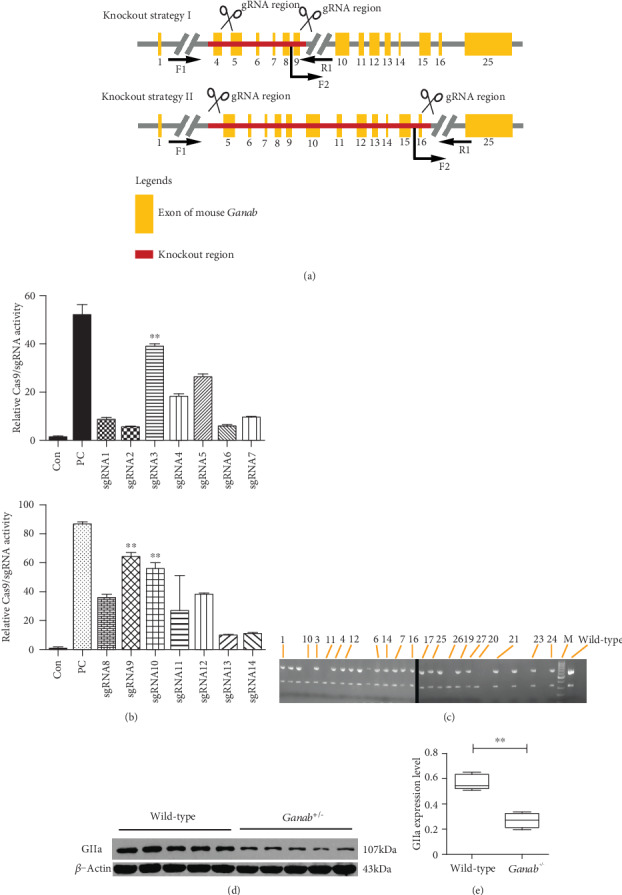
*Ganab*-haploinsufficient mice were constructed via two strategies. (a) *Ganab*^+/-^mouse construction strategies. Strategy I, the designed sgRNA was located in the nonconserved region between intron 4 and intron 10. Strategy II, the designed sgRNA was located in the nonconserved region between intron 5 and intron 17. (b) Screening of sgRNA in targeting vectors, and sgRNA3 and sgRNA9 with higher viability were selected for efficient *Ganab* gene knockout. (c) Genotyping was performed on randomly selected mice (wild-type and mutant mice) to confirm the absence of the *Ganab* gene in the mutant mice. Four homozygotes were found in 3.5-day mouse embryos constructed by strategy II. PCR product size for homozygotes: 622 bp/335 bp; heterozygotes: 622 bp/658 bp/335 bp; wild-type allele: 658 bp/335 bp. (d, e) Compared with that in the wild-type group, the GIIa protein expression in the *Ganab*^+/-^ group (strategy I) was significantly reduced, and the difference was statistically significant (*n* = 5). Data are presented as the mean ± SEM. ^∗∗^*P* < 0.001 by 2-tailed *t* test.

Based on sgRNA design principles, in Genotyping strategy I (Figure 1(a)), 7 sgRNAs were designed in the nonconserved regions in intron 4 and intron 10. Based on the results of these 14 sgRNA activity tests, sgRNA3 and sgRNA9 were identified as candidates. The sgRNA3 sequence is CTGGCCCTAAAATCAAGCCTTGG, and the sgRNA9 sequence is AGGACTTCGGGAGTGGTAAATGG. Similarly, the candidate sgRNA sequences in Genotyping strategy II are TGCAGTGCTACCAATTCATCTGG and CCTGCCAGAAGGCTTAAGCGAGG, respectively.

### 2.3. Immunofluorescence Staining

The tissues were placed in a 4% paraformaldehyde (MilliporeSigma) solution and placed in a 4°C refrigerator overnight. The tissues were placed in 10% sucrose/PBS, 20% sucrose/PBS, and 30% sucrose/PBS at 4°C for 4 hours, 6 hours, and 4 hours, respectively. The tissue was placed in a frozen mold and fixed in OCT. The frozen tissue was sectioned (4 *μ*m thick) and permeated using 0.05% Triton X-100 in PBS, pH 7.4. Sections were blocked with 0.1% casein (Vector Labs). Primary antibodies (diluted with 0.1% casein) were added dropwise and incubated in a 4°C refrigerator overnight. After sufficient washing, the samples were incubated with the appropriate fluorescent secondary antibody (1 : 400 dilution) for 1 hour at room temperature and counterstained with DAPI-containing mounting slides. A scanning confocal microscope (Olympus FluoView 1000) was used to capture each fluorescence channel separately.

### 2.4. Micro-CT and Angiography

To better observe the blood vessels in the kidneys of mice, we injected ExiTron™ nano 12000 CT contrast agent (order no. 130-095-698) into the tail veins of the mice. Each mouse was injected with 170 *μ*L. After injection of the contrast agent, the mice were placed in a box for anesthesia. After the anesthesia was sufficient, the mice were placed in a micro-CT (Source-Ray SB-80-1K, Varex Imaging, USA) scanner and scanned to obtain a mouse kidney angiogram.

### 2.5. Primary Cilia Length Measurement

Frozen sections (4 *μ*m thick) of kidney tissue were incubated with an anti-*α*-acetylated tubulin antibody (T6793, Millipore Sigma) at 4°C overnight and then incubated with secondary antibodies for 1 hour at room temperature. After washing, the sections were counterstained and mounted with DAPI-containing mounts. A scanning confocal microscope (Olympus FluoView 1000) was used to capture each fluorescence channel separately. ImageJ software (NIH, https://imagej.nih.gov/ij/) was used to measure the length of the primary cilia.

### 2.6. Western Blotting

Western blotting was performed as previously described [6]. The isolated mouse kidney was washed with ice-cold PBS, pH 7.4, and lysed with ice-cold RIPA lysis buffer for 1 hour at 4°C. The samples were centrifuged at 12,000 g for 15 minutes at 4°C. The samples were subjected to SDS-PAGE, transferred to PVDF membranes, and detected with appropriate primary antibodies and then with HRP-conjugated anti-mouse, anti-goat, or anti-rabbit IgG. SuperSignal West Dura Extended Duration Substrate (Pierce) was used to detect the blot signal by enhanced chemiluminescence. Bands were detected by enhanced chemiluminescence, and relative protein expression levels were quantified using ImageJ.

### 2.7. Blood Pressure Measurement in Mice

One week before the data were officially recorded, the blood pressure of the mice was measured multiple times at a fixed time to accommodate the stimulus. The tail cuff was placed around the root of the mouse's tail when measuring. Before measuring the blood pressure, we checked whether the cuff was leaking, and leaking cuffs were replaced; the data were recorded at the stage where the animal's heartbeat was stable, and the average value was determined from repeated measurements.

### 2.8. Statistics

Data that conformed to a normal distribution were analyzed using a *t* test and analysis of variance, and data that did not conform to a normal distribution were analyzed using the rank-sum test. *P* < 0.05 represents statistical significance, and the data in the figures are expressed as the mean ± SEM. SPSS statistical software (version 25.0) was used.

## 3. Results

### 3.1. *Ganab-*Haploinsufficient Mice Were Constructed via Two Strategies

The *Ganab* gene (NCBI ID: 14376) is located on the positive strand of mouse chromosome 19 and has a full length of 18.7 kb. To increase the likelihood of generating a *Ganab* knockout, we designed two knockout strategies. In strategy I, the designed sgRNA was located in the nonconserved region between intron 4 and intron 10. In strategy II, the designed sgRNA was located in the nonconserved region between intron 5 and intron 17 (Figure 1(a)). The activity of sgRNA in the targeting vector was detected, and sgRNA 3 and sgRNA 9 with higher viability were selected, ligated to the promoter plasmid vector and transcribed in vitro to obtain microRNA for microinjection (Figure 1(b)). To test whether our models were successfully constructed, we designed primers and performed PCR for genotyping and found that they were all either wild-type (*wild-type*) or heterozygous (*Hete*). To obtain homozygous (*Homo*) mice, we screened the embryos of the model mice, which were constructed by strategy II. We detected the *Homo* genotype in 3.5-day embryos (Figure 1(c)), while no *Homo* genotype was detected in embryos after 3.5 days or in postnatal mice (Table S1). Genotyping of 27 embryos (3.5 days) was performed and identified 15 heterozygotes (*Ganab*^+/-^) and 4 homozygotes (*Ganab*^−/−^). A total of 128 offspring were examined from postnatal days 1 to 21, of which, 92 were *Ganab*^+/-^, and we observed no homozygotes in the offspring. These observations suggest that the *Ganab* gene may be involved in early embryonic development, and homozygous *Ganab* gene deletion is embryonic lethal. To examine *Ganab* expression in the mouse kidney, we used Western blotting to measure the abundance of the glucosidase II alpha subunit. Compared with the *wild-type* group (*Ganab*^+/+^), the *Ganab*^+/-^ group showed significant downregulation of glucosidase II alpha (*GIIa*) subunit expression, and the difference was statistically significant (*P* < 0.05), indicating that heterozygous mutation or haploinsufficiency of *Ganab* significantly affected the expression of the glucosidase II alpha subunit (Figures 1(d) and 1(e)).

### 3.2. No Kidney or Liver Cyst Phenotypes Were Observed in *Ganab*^+/-^ Mice

**Figure 2:**
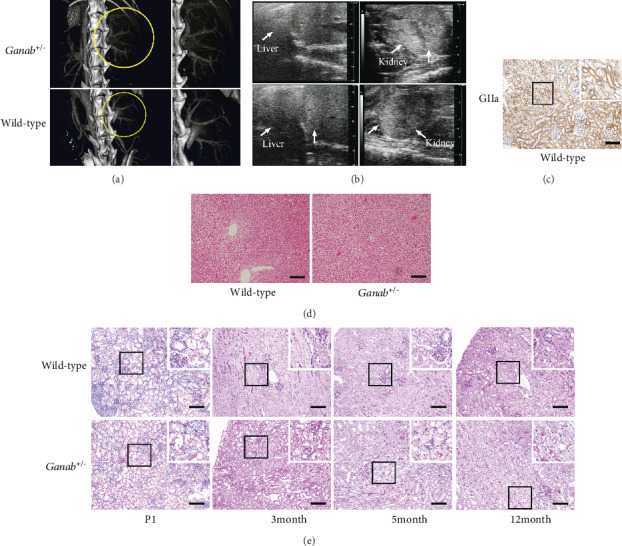
No kidney or liver cyst phenotypes were observed in *Ganab*^+/-^ mice. (a) There were no obvious abnormalities in blood vessels and tissues of *Ganab*^+/-^ mice via angiographic contrast-enhanced CT examination (*n* = 5). (b) No liver or kidney cysts were found in the *Ganab*^+/-^ group by Doppler ultrasonography (*n* = 5 per group). (c) Immunohistochemical staining of wild-type kidney tissue with anti-GIIa antibody showed that GIIa was mainly distributed in the renal tubules. (d) H&E staining of liver tissues showed that no cysts were observed in the *Ganab*^+/-^ or wild-type liver. (e) PAS staining of kidney tissues from P1 to 12 months showed that no obvious dilated tubules were observed in *Ganab*^+/-^ and wild-type kidneys (*n* = 5). Scale bars: 100 *μ*m.

To determine whether the *Ganab*^+/-^ mice had arterial defects, especially in the renal arteries, the mice were injected with the nano 12000 contrast agent and examined via micro-CT. The results revealed that there were no obvious arterial or venous abnormalities in the *Ganab*^+/-^ mice (Figure 2(a)). To examine whether the *Ganab*^+/-^ mice that we constructed developed kidney or liver cysts similar to human patients, we performed Doppler ultrasonography examination on the animals. We failed to detect any kidney or liver cysts in either of the two strains (Figure 2(b) and Figure S1B). To determine the specific expression pattern of the *Ganab* gene in the kidneys of *wild-type* mice, we performed immunohistochemical staining of paraffin sections. We found that the glucosidase II alpha subunit was mainly distributed in the renal tubules of the kidney, while it was absent in the glomeruli and intrarenal blood vessels (Figure 2(c)). H&E staining of liver tissues showed that no cysts were observed in *Ganab*^+/-^ or wild-type groups (Figure 2(d)). Clinically, imaging techniques, such as Doppler ultrasound, CT, and MRI, are used to observe the size, number, and morphological changes of kidney cysts. However, although immunohistochemical staining suggested that glucosidase II alpha (GIIa) was expressed in the renal tubules, we did not detect tubular dilatations in the kidneys of *Ganab*^+/-^ embryos or mice up to 12 months old with PAS staining (Figure 2(e)). We measured the blood flow and structure of the renal arteries and veins in mouse kidneys via Doppler ultrasound examination. The results showed no significant abnormalities in the *Ganab*^+/-^ mice. We also measured the blood pressure in the *Ganab*^+/-^ mice and did not obtain any abnormal findings (Table S2).

### 3.3. The Primary Cilia and the Expression of PC1 and PC2 Were Not Affected in the *Ganab*^+/-^ Mice

**Figure 3:**
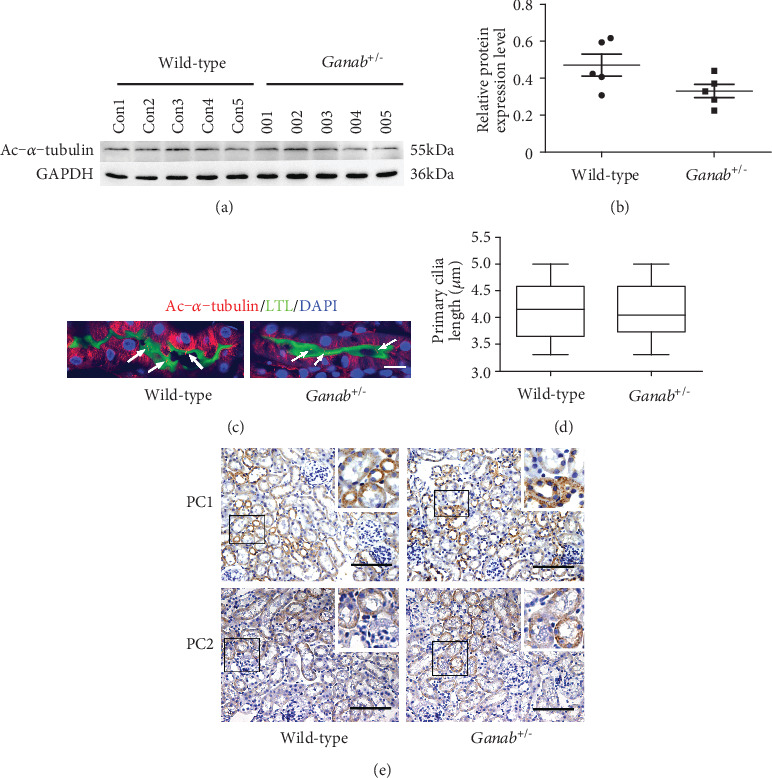
The primary cilia and the expression of PC1 and PC2 were not affected in the *Ganab*^+/-^ mice. (a, b) Ac-*α*-tubulin was expressed in both the wild-type group and the *Ganab*^+/-^ group (a), and there was no significant difference between them (*P* = 0.078) (b). Data are presented as the mean ± SEM. *P* > 0.05 by 2-tailed *t* test. (c, d) Primary cilia stained with anti-Ac-*α*-tubulin (red) are shown in the *Ganab*^+/-^ group and the wild-type group (c) and showed no significant difference in the length of the primary cilium between the *Ganab*^+/-^ group and the wild-type group (d) (*n* = 5). Data are presented as the mean ± SEM. *P* > 0.05 by 2-tailed *t* test. (e) The expression of PC1 and PC2 protein in the *Ganab*^+/-^ group was not significantly different from that in the wild-type group (*n* = 5). Data are presented as the mean ± SEM. *P* > 0.05 by 2-tailed *t* test. Scale bars: 100 *μ*m.

To detect whether primary cilia (which play a key role in the development of ADPKD renal cysts) are affected in the *Ganab*^+/-^ mice, we examined the specific marker of primary cilia, acetylated tubulin (Ac-*α*-tubulin), and Western blot results showed that Ac-*α*-tubulin was expressed in both the *wild-type* group and the *Ganab*^+/-^ group (Figure 3(a)), and the difference was not significant (*P* = 0.457) (Figure 3(b)). Immunofluorescence staining was also performed to observe primary cilia length (Figure 3(c)). The results showed no significant difference in the length of the primary cilia between the *Ganab*^+/-^ group and the *wild-type* group (Figure 3(d)). To observe whether *Ganab* haploinsufficiency affects the expression of PC1 and PC2 proteins, we performed immunohistochemical staining (Figure 3(e)). The expression of PC1 and PC2 proteins in the *Ganab*^+/-^ group was not significantly different from that in the *wild-type* group. This result indicated that the expression of PC1 and PC2 was not significantly affected when the *Ganab* protein was reduced in *Ganab*^+/-^ kidneys.

## 4. Discussion

Glucosidase II, which is located in the endoplasmic reticulum, consists of the *α* catalytic subunit and a *β* subunit, the former of which is encoded by the *GANAB* gene [7]. The main function of glucosidase II is to promote protein folding by catalyzing the hydrolysis of glucose residues by immature glycoproteins. Defects in the *α* catalytic subunit result in protein misfolding. The misfolded proteins are then reglycosylated by UDP-glucose glycoprotein glucosyltransferase (UGGT), which causes the secreted proteins to remain in the calcitonin and calmodulin cycle for an extended period of time. This process can potentially cause defects in the maturation of the polycystin-1 and polycystin-2 proteins. In silico and RT-PCR analyses show that the *GANAB* gene is expressed approximately equally in the human kidneys and liver, that the *GANAB* protein mainly plays a role in assisting PC1 and PC2 protein maturation, and that *GANAB* mutations cause kidney and liver cysts via loss of these functions [3, 8]. Therefore, mutations in the *GANAB* gene can cause ADPKD and polycystic liver disease. These potential pathologies are consistent with the previously reported familial findings. Haploinsufficient genes, such as the *PKD1* and *PKD2* genes, cause insufficient protein production and are not sufficient for normal function [9, 10].

In this experiment, the GIIa protein expression of the *Ganab* gene in mice constructed by the two strategies decreased by more than 50% (Figure S1A), and no cysts were seen in the mice after birth (Figure S1B). This shows that the phenotypes of mice constructed by these two strategies are not significantly different. Therefore, this study mainly chose the mouse models constructed by strategy II for embryo testing. Although we found that GIIa protein expression of the *Ganab* gene decreased by more than 50% in the *Ganab*^+/-^ kidney, PC1 and PC2 protein expressions were not affected. At the same time, primary cilia are generally considered to be closely related to the pathogenesis of polycystic kidney disease, but deletion of the *Ganab* gene in this experiment did not affect the length of the primary cilia. In addition, previous studies have suggested that the occurrence of cysts in ADPKD patients also involves arterial blood vessels. Therefore, we performed angiographic examination and blood pressure measurement on *Ganab*^+/-^ mice, but compared with those of the control group, the vascular morphology and blood pressure of the mutant group were normal. Generally, GIIa is mainly expressed in the renal tubules of the kidney tissue. If the inadequate function of the haploid *Ganab* gene affects the expression of GIIa, theoretically, the phenotype should first appear in the renal tubules. However, in this experiment, due to the knockdown of the *Ganab* gene, the expression of GIIa decreased, and no renal tubular abnormalities, such as cystic dilatation and primary cilia damage, were observed. Based on the above results, we believe that a simple heterozygous mutation of the *Ganab* gene alone will not affect the expression of the *Pkd1* and *Pkd2* genes; in addition, unlike the heterozygous *Pkd1* and *Pkd2* mice with obvious cysts at 3 months [11], mice heterozygous for the *Ganab* gene, also known as *Pkd3*, had no cysts. Because kidney samples from patients have never been included in the study of the *GANAB* gene and *Ganab*^+/-^ animal models have never been constructed before, the mouse kidney tissue constructed in this study is crucial for studying the role of this gene. However, *Ganab* haploinsufficiency does not cause kidney or liver cysts in mice.

## 5. Conclusions

The main purpose of this study was to study the role of the *Ganab* gene in the first animal model constructed. Previous studies have suggested that the *GANAB* gene plays an important pathogenic role in ADPKD. Due to the lack of kidney samples from patients with this gene mutation and animal models that do not yet have this gene, we constructed a mouse model of the *Ganab* gene mutation. In this study, two targeting strategies were established using CRISPR/Cas9 technology, and a *Ganab* knockout mouse model was constructed. The results obtained confirmed that homozygous mutations in the *Ganab* gene are early embryonic lethal, while the haploid function caused by the heterozygous *Ganab* mutation is insufficient to cause kidney and liver cysts in mice. Therefore, we cautiously believe that, at least in mice, haploinsufficiency of the Ganab gene will not be sufficient to cause cysts.

## Data Availability

The data used to support the findings of this study are available from the corresponding author upon request.

## Supplementary Materials

Supplementary materials Materials and methods. Table S1: partial embryo PCR validation results. Table S2: blood pressure measurement in mice. Figure S1: Ganab-haploinsufficient mice constructed via two strategies can result in decreased protein expression and no cysts in the kidney.
