# Supplementary material for: Ganab Haploinsufficiency Does Not Cause Polycystic Kidney Disease or Polycystic Liver Disease in Mice
Source: Biomed Res Int. 2020 May 19;2020:7469428. doi: 10.1155/2020/7469428 (PMC7256702; doi:10.1155/2020/7469428)
Supplement: Supplementary materials — Materials and methods. Table S1: partial embryo PCR validation results. Table S2: blood pressure measurement in mice. Figure S1: Ganab-haploinsufficient mice constructed via two strategies can result in decreased protein expression and no cysts in the kidney. [file 7469428.f1.docx]

Supplementary Materials for

*Ganab* haploinsufficiency does not cause polycystic kidney disease or polycystic liver disease in mice

Guangrui Geng^1^, Yunming Xiao^1^, Yingjie Zhang^1^, Wanjun Shen^1^, Jiaona Liu^1^, Fei Zhu^1^, Xu Wang^1^, Jie Wu^1^, Ran Liu^1^, Guangyan Cai^1^, Qinggang Li^1^, Xueyuan Bai^1^, Xiangmei Chen^1^

^1^Department of Nephrology, Chinese PLA General Hospital, Medical School of Chinese PLA, Chinese PLA Institute of Nephrology, State Key Laboratory of Kidney Diseases, National Clinical Research Center for Kidney Diseases, Beijing, China.

The PDF file includes:

Materials and methods

Table S1. Partial embryo PCR validation results

Table S2. Blood pressure measurement in mice

Materials and Methods

***Antibodies.*** The primary antibodies included anti-GIIa (ab96757, Abcam), anti-Polysystin 1 (catalog sc-130554, Santa Cruz Biotechnology), anti-Polysystin 2 (catalog sc-47734, Santa Cruz Biotechnology), anti-Beta Actin (catalog 60008-1-Ig, Proteintech), fluorescein-conjugated LTL (catalog FL1321, Vector Labs), anti–α-acetylated tubulin (catalog T6793, Millipore Sigma). The secondary antibodies included HRP-conjugated anti-goat, anti-mouse, and anti-rabbit antibodies (catalog A0181, A0216, and A0208, Beyotime); FITC-conjugated AffiniPure donkey anti–goat IgG (H + L) (Jackson ImmunoResearch); Cy3-conjugated AffiniPure donkey anti–goat IgG (H + L) (Jackson ImmunoResearch); FITC-conjugated AffiniPure donkey anti–rabbit IgG (H+L) (Jackson ImmunoResearch); and Cy3-conjugated AffiniPure donkey anti–rabbit IgG (H + L) (Jackson ImmunoResearch).

***Relevant Reagents.*** Trizma Hydrochloride Solution (Cat. No. T2663, Sigma), Proteinase K (Cat. No. MK539480, Merck), Triton X-100 (Cat. No. T8787, Sigma), 2×Taq Master Mix (Dye Plus) (Cat. No. P112-01, Vazyme), Agarose (BIOWEST AGAROSE, REGULAR), DNA Marker (100bp DNA Ladder #SM0242, Thermo Scientific GeneRuler), 0.5×TBE (Cat. No. 0105-500g, EDTA Shanghai Sangon).

***DNA Extraction.*** A low-cost and sample method to gain rough genomic DNA.

**(a)** Add 100 μL of tail digestion buffer per tail piece (2-5 mm) in a microcentrifuge tube. Be careful not to cut too much tail. **(b)** Incubate the tube at 56℃ overnight. **(c)** Incubate the tube at 98℃ for 13 minutes to denature the Proteinase K. **(d)** Spin in microcentrifuge at top speed for 15 minutes. Use an aliquot of supernatant straight from the tube (2 μL in a 50 μL reaction) for PCR. Final concentration of tail digestion buffer: 50 mM KCl; 10 mM Tris-HCl (pH 9.0); 0.1 % Triton X-100; 0.4 mg/mL Proteinase K.

***PCR Conditions Attachment.*** PCR was carried out in 25 µL volume for 35 cycles under standard conditions, with primers listed above added to each reaction. Taq DNA polymerase used was P112-01. Two controls used in PCR genotyping: No DNA template added in Water control and 400 ng of mouse genomic DNA in Wildtype control.

Table S1. Partial embryo PCR validation results

| Gestational | 3.5d | 12.5d | 16.5d |
| --- | --- | --- | --- |
| Parent information | F2-1#*F3-6#,F3-7 | F2-8#*F2-18# | F2-1#*F2-5# |
| Total number of embryos | 27 | 8 | 7 |
| Genotype and numbers | (-/-): 10, 11, 20, 26 ;  (+/-): 1, 3, 4, 6, 7, 12, 14, 16, 17, 19, 21, 23, 24, 25, 27 | (+/-): 1, 2, 3, 4, 5, 6, 7, 8 | (+/-): 3, 4, 5 |

Table S2. Blood pressure measurement in mice

| Mouse ID | SBP | DBP | MAP | HR |
| --- | --- | --- | --- | --- |
| 1E2Q8-001 | 117.642 | 83.534 | 94.903 | 503 |
| 1E2Q8-005 | 121.621 | 95.222 | 104.022 | 624 |
| 1E2Q8-007 | 123.325 | 87.156 | 99.212 | 610 |
| 1E2Q8-008 | 126.23 | 82.245 | 96.907 | 465 |
| 1E2Q8-009 | 137.815 | 92.482 | 107.593 | 501 |
| 1E2Q8-010 | 123.147 | 89.375 | 100.632 | 524 |
| 1E2Q8-014 | 114.42 | 86.21 | 95.613 | 433 |
| 1E2Q8-019 | 116.206 | 71.134 | 86.158 | 421 |
| 1E2Q8-020 | 117.281 | 83.246 | 94.591 | 508 |
| 1E2Q8-021 | 122.227 | 83.312 | 96.284 | 541 |
| 1E2Q8-024 | 98.632 | 64.514 | 75.887 | 415 |
| Control-1 | 118.73 | 75.378 | 89.829 | 411 |
| Control-2 | 115.864 | 72.185 | 86.745 | 433 |
| Control-3 | 127.034 | 83.352 | 97.913 | 519 |
| Control-4 | 125.483 | 92.432 | 103.449 | 499 |
| Control-5 | 124.788 | 91.334 | 102.485 | 486 |


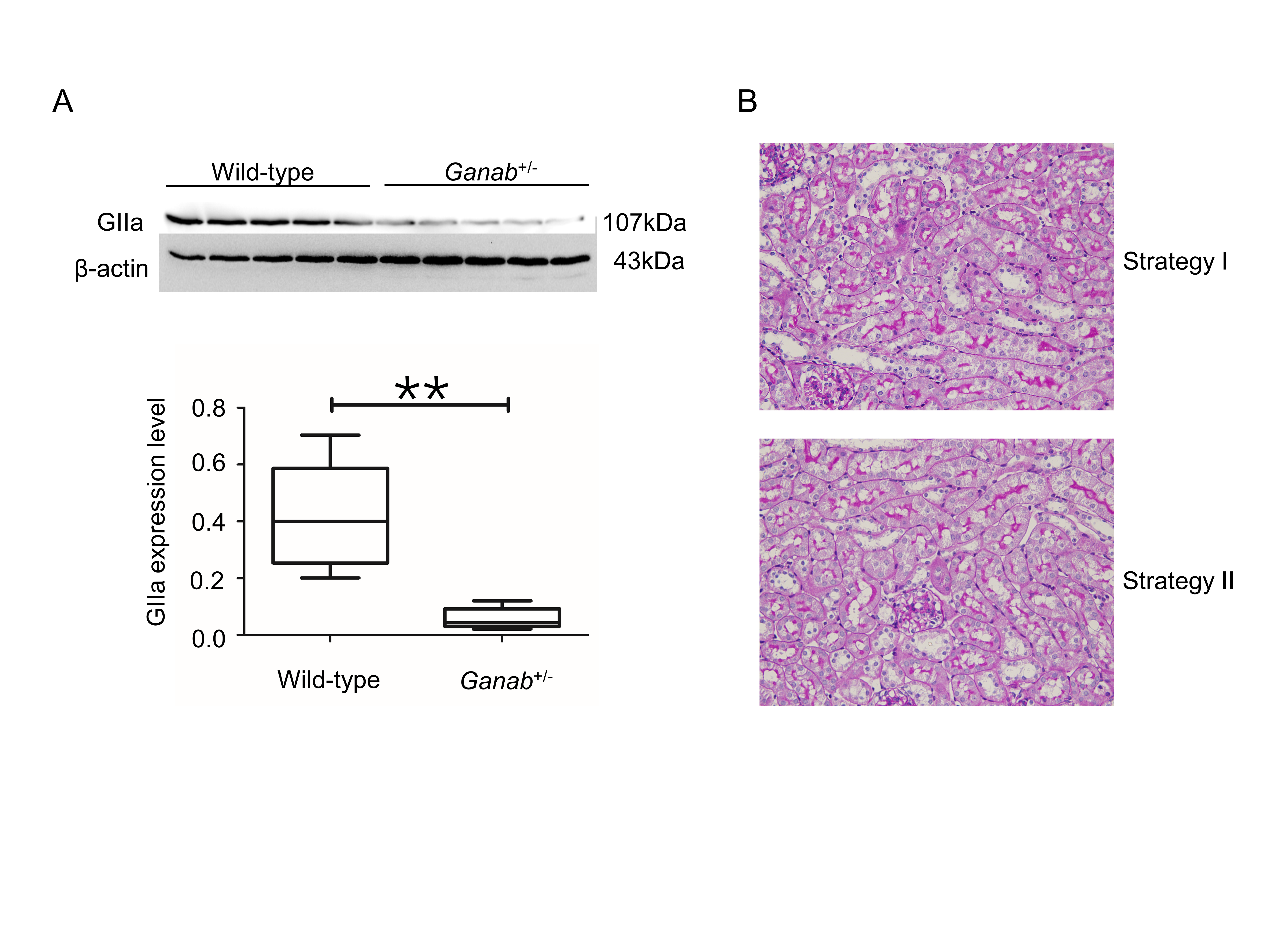


**Figure S1. *Ganab*-haploinsufficient mice constructed via two strategies can result in decreased protein expression and no cysts in the kidney.** (A) Compared with that in the wild-type group, the expression of GIIa protein in mice constructed by Strategy II was significantly reduced, and the difference was statistically significant (n = 5, P=0.003). Data are presented as the mean ± SEM. **P < 0.001 by 2-tailed t test. (B) The symptoms of mice constructed by the two strategies were not significantly different, and no cysts were found in their kidneys.
